# Developing power plant materials using the life cycle lens

**DOI:** 10.1098/rsta.2023.0409

**Published:** 2024-10-09

**Authors:** Amanda Quadling, David Bowden, Chris Hardie, Arti Vasanthakumaran

**Affiliations:** ^1^ Materials Division, UK Atomic Energy Authority, Culham Science Centre, Abingdon, Oxfordshire OX14 3DB, UK

**Keywords:** reduced activation steels, crystal plasticity, FISPACT-II

## Abstract

The Spherical Tokamak for Energy Production (STEP) environment will include magnetic, thermal, mechanical and environmental loads far greater than those seen in the Joint European Torus campaigns of the past decade or currently contemplated for ITER. Greater still are the neutron peak dose rates of 10^−6^ displacements per atom, per second, which in-vessel materials in STEP are anticipated to be exposed to. Reduced activation and high-fluence resilience therefore dominate the materials strategy to support the STEP Programme. The latter covers the full life cycle from downselected compositions and new microstructural developments to irradiation-informed modelling and end-of-life strategies. This article discusses how the materials downselection is oriented in plant power trade-off space, outlines the development of an advanced ferritic-martensitic structural steel, describes the ‘Design by Fundamentals’ mesoscale modelling approach and reports some of the waste mitigation routes intended to make STEP operations as sustainable as possible.

This article is part of the theme issue ‘Delivering Fusion Energy – The Spherical Tokamak for Energy Production (STEP)’.

## 1. Introduction

For some time now, fusion engineers have understood that confinement devices present formidable challenges to materials [1]. However, the Spherical Tokamak for Energy Production (STEP) Programme work of the past 3 years has highlighted that in moving from device to power plant, there must be a significant extension in the materials scientist’s priorities beyond the traditional focus on neutron resilience of first wall and heat dissipation in tokamak divertors, for example. Fuel cycle (including tritium breeding, mobility and inventory) input and power output considerations now dominate, alongside plant availability and reliability and the vexed question of qualification of materials in an entirely new operating regime, currently unavailable to developers.

Choices of breeder and coolant impact the parasitic power loads required to circulate both, and this in turn dictates downselection of compatible blanket substrates. The thermal capabilities of the latter must optimize heat exchanger transfers to the BoP. Hence, the STEP materials strategy has focused heavily on *high temperature structural steels development*. For qualification (or at least steps towards performance assurance), design engineers require First-of-a-Kind (FOAK) mesoscale models, employing solid-state physics to predict the evolution of polycrystalline microstructures under neutron irradiation. The STEP materials team has thus embarked on a broader *assurance strategy* around irradiations, subsequent testing and data feed to *crystal plasticity models*. Plant performance revolves around component lifetimes which can be tuned using *state-of-the-art inventory codes* to determine rates of materials degradation (by displacement and/or transmutation) but also the rate of activation of materials in service. Hence, the STEP materials strategy has focused strongly on *active waste mitigation* through materials choice and materials engineering.

This article will provide some detail on each of these three areas of high-temperature steels development, crystal plasticity modelling and active waste mitigation strategies. Emerging from the above is a full life cycle lens to materials R&D, with STEP researchers considering design and development, through irradiation and assurance, to routes to replacement and disposal of neutron-exposed materials. To contextualize the above, a brief overview is provided on the trade space for materials within a typical tokamak-based powerplant.

## 2. Approaching the STEP tokamak

At the most basic level, the STEP irradiated tokamak environment can be reduced to eight key materials needs (armour, breeder, tritium barrier, structural, shielding, magnet, coolant carrier/heat conductor and electronics) in three broad engineering set pieces:

— The inboard area around the central solenoid requires a magnet, shielding, coolant-carrying pseudo-structural and first-wall armour.
— The divertor requires magnet, shielding, coolant carrier and high heat flux armour.
— The outboard area requires a neutron transparent first-wall armour, breeder, structural, tritium barrier for firewall to the balance of plant (BoP) and potentially, a lithium barrier.

Radhard electronics for diagnostics and controls are needed throughout. Fluences at the vacuum vessel are considered low enough to proceed with standard industrial steels. In the early months of the Programme, a multi-disciplinary team evaluated various permutations of magnet and coolant regime, specifically to consider the degree of interdependency of materials choices. The exercise focused on shielding, structurals and heat conductors. Some indicative trade-offs are presented for interest:

*Scenario 1: Cryogenically cooled aluminium magnets + supercritical CO*_*2*_ *coolant*

— BoP ideally requires >450°C temperatures for economically viable thermo-electric conversion using a sCO_2_ coolant but a pebble breeder can’t exceed 550°C based on current structural material capabilities under neutrons [2,3]. The 350–550°C possible window of operation is quite constrained.
— A sequence of thermal screens (multi-layers which include intermediate conduction shields, cooling vapour shields and highly reflective materials) will be needed between the cryogenic cooling circuit of the magnet and the high heat fields of the vessel. Some of these materials are known to be difficult to outgas [4,5].
— At cryogenic temperatures, various materials’ thermal conductivity and specific heat capacity are probably to deteriorate precisely at the time when neutron heating of the magnet materials most implies the need for heat dissipation [6,7]. There is a challenge for materials interfaces to prevent potential thermal runaway.

*Scenario 2: FLiBe coolant and low field strength (ductile) superconducting magnets*

— FLiBe as a coolant candidate offers high breeding ratios but will produce fluorine-boosted corrosion [8,9]. The latter implies a need for nickel-based alloys as corrosion coatings in the coolant containment (but the latter results in high activation-induced swelling and high magnetic susceptibility) [10]. Alternative coating material to the Ni-based alloys could be boron nitride, but the large neutron cross-section of the boron counts against the breeding function [11]. Serious reactions with air/water dictate a stringent separator requirement between the FLiBe as primary coolant and water as secondary coolant, in the heat exchanger. The former restricts design options and constrains the use of additive manufacturing.
— To keep the FLiBe molten, the higher lithium eutectic temperature of 460°C is required as a minimum temperature in the system [12]. Relative to the irradiated steels cap at 550°C, the operating window for the integrated blanket/wall system becomes extremely limited.
— For power generation, the benefit of FLiBe’s higher boiling point (>1000°C) must be contrasted with the corrosion impact it delivers as temperatures rise and the creep impact to metals (suggesting a more realistic operating benefit between 600 and 700°C) [13,14]. The latter would imply the use of SiC_f_–SiC structural materials (for example) instead of a steel.
— Because the superconducting magnets require more shielding (than cryogenically cooled aluminium magnet coils), thought is needed about the central column interface layers—most probably stainless steel [15]. In this high-temperature region, the nickel in stainless steel (e.g. ss316) will strongly activate [16].

*Scenario 3: Liquid metal coolant and high field strength (brittle) superconducting magnets*

— A minimum temperature is required in order to circulate the liquid metal coolant, and this sits generally at approx. 200°C for most options (liquid lead and liquid lead lithium eutectic), reducing the head room at the powerplant end (relative to gas cooling options) and requiring power inputs to lag/heat pipework [17]. Liquid metal coolant top temperatures are >1000°C, suggesting that if it were possible to move past the 550°C fusion steels constraint, more flexibility and power would open up [18].
— Because of the high breeding ratios in the liquid metal but the non-absorption of neutron energy by lead, greater shielding will be required in the coolant containment [19]. If selecting tungsten carbides, thought will need to be given to the decay heat generated near cameras in the monitoring/ diagnostic pieces.
— Because of the response of the liquid metal to the magnetic fields, flow will be limited unless flow channel inserts are used (SiC worth further investigation for this environment and functionality) and/or vanadium for magnetic insulation. Alumina and other ceramics will also reduce the magnetohydrodynamic effects on the liquid metal coolant but will require irradiation resilience investigations [20]. Ceramics are less well understood with respect to neutron damage, relative to the metals.

While decisions around STEP magnets increasingly focus on high-temperature superconductors and breeder–coolant choices remain in development, a commonly emerging theme from the scenario evaluations is the need for a high-temperature structural material. In this regard, in addition to steels development, the materials support for STEP has included consideration of ceramic composite materials and metal alloys of vanadium. For the latter, structural compromises around creep resilience might be offset against better resistance to neutrons in proximity to liquid metal or lithium-based breeder–coolants. All three avenues are being explored. Early work on fusion steel is summarized below, followed by assurance methodology development as well as waste strategy considerations—both pertinent to future steels and other STEP materials.

## 3. High-temperature structural steels development

**Figure 1.**
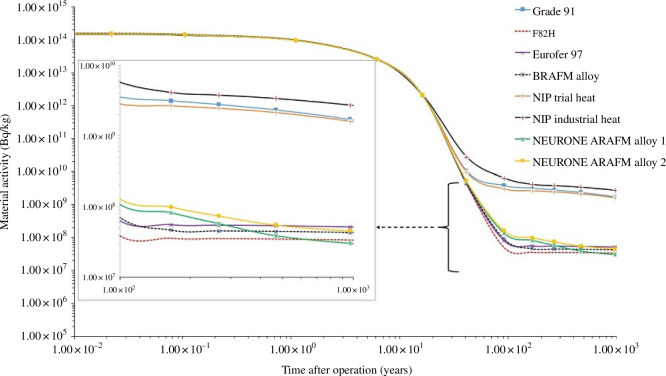
Activity (Bq/kg) versus decay time after neutron exposure ceases (years) for a range of steels within the mid-blanket region. Steels such as grade 91 and the Nuclear Innovation Programme (NIP) heats were not reduced activation and this difference in increased activity is clearly demonstrated when compared with RAFM alloys such as Eurofer97, F82H and the ARAFM and BRAFM alternatives being developed.

Globally, fusion steels interest revolves around oxide dispersion strengthened variants as well as reduced-activation ferritic-martensitic (RAFM) steels. International efforts in the latter have focused on producing large-scale production of ingots designed to offer reduced levels of radioactivity, enabling re-processing of the steel as low-level waste (LLW) within 100 years after the steel is removed from the plant [21]. Within the European Union, Eurofer97 is the primary RAFM alloy of choice for breeder and divertor structurals, with four large tonnage-scale heats having been produced to date. The typical composition of Eurofer97 is compared with commercial grade 91 steel in table 1 and figure 1. In particular, in Eurofer97 the concentrations of Ni, Mo, and Nb are eliminated or reduced for activation purposes in favour of W, Ta and V [25]. W provides solution strengthening, while Ta and V act as C-getters to form fine carbide/nitride precipitates for added strength. B has also been introduced previously as a grain boundary stabilizer for enhanced high-temperature resistance method to stabilize M_23_C_6_, preventing excessive coarsening at high temperatures.

**Table 1.** Typical composition of grade 91 steel in comparison to RAFM grades such as Eurofer97, F82H and the ARAFM and BRAFM derivatives being developed in the UK. All values are shown in wt%.

Alloy	Cr	Ni	W	Mn	Mo	Nb	Ti	V	Ta	Si	C	B	N	Fe
Grade 91 [22]	8–9.5	0–0.4	—	0.3–0.6	0.85–1.05	0.06–0.1	0–0.01	0.18–0.25	—	0.2–0.5	0.08–0.12	—	0.03–0.07	Bal.
Eurofer97 (ideal) [23]	8.5–9.5	0–0.01	1.0–1.2	0.2–0.6	0–0.005	0–0.005	0–0.02	0.15–0.25	0.10–0.14	0–0.05	0.09–0.12	0–0.002	0.015–0.045	Bal.
F82H (ideal) [24]	7.5–8.5	—	1.6–2.2	0.05-0.5	—	—	0–0.01	0.15–0.25	0.02–0.1	0–0.02	0.08–0.12	0–0.006	0–0.025	Bal.
NEURONE ARAFM	8–9	—	2	0.07–0.4	—	—	0–0.07	0–0.3	0.07–0.1	0.05–0.1	0.07–0.1	—	0–0.025	Bal.
BRAFM	7.5–9	—	1.2–2	0.25	—	—	0–0.02	0–0.25	0.07–0.14	0.1–0.5	0.1–0.13	0–0.015	0.01–0.015	Bal.

The range of damage types that these steels experience in service is variable, and temperature dependant. This damage evolves as a combination of radiation-induced effects, thermal loading and in the case of creep, mechanical loading. It is well documented that Eurofer97 possesses poor creep performance at a baseline 100 MPa at temperatures exceeding 550°C [26]. Hence, this temperature is often cited as the maximum operating temperature of Eurofer97 and other conventional RAFM steels in a fusion environment. Embrittlement of steels occurs at irradiation temperatures below 350–400°C [27,28], referred to as low-temperature hardening embrittlement (LTHE). Such embrittlement effects are manifested through the formation of vacancy and interstitial defect clusters, as well as the segregation of elements like manganese and silicon. Above approx. 550°C, helium, a decay product arising primarily from (n, α) reactions with iron, accumulates at the grain boundaries, leading to high-temperature helium embrittlement [29].

Steels development efforts for STEP have focused on expanding the operating temperature window for advanced (A)RAFM steels, aiming to increase the capacity to withstand creep effects at temperatures exceeding 550°C, while decreasing the propensity to embrittle and subsequent loss of toughness when irradiated below 400°C and managing problematic decay products, such as helium [30–32]. The primary strengthening mechanism utilized in these advanced steels are nanoscale precipitates, namely carbides and nitrides, explored for high-temperature nuclear steels previously [25]. Two key precipitates are utilized, the variants MX and M(Cr)_23_C_6_. MX precipitates are formed throughout the microstructure, including at intragranular sites and can pin defects arising from irradiation and plasticity-induced damage, while acting as sinks for mobile elements, such as transmutation gases [33]. Metallic (M) elements such as vanadium, titanium or tantalum are used to form MX, while carbon or nitrogen comprise the X element. The M_23_C_6_ form predominantly at lath and prior-austenite boundaries within the martensitic matrix, and restrict lath boundary sliding, further impeding high-temperature creep [34]. These carbides comprise primarily Cr as the M element, although these carbides can commonly occur in a mixed form, usually alongside Fe. M_23_C_6_, however, must be carefully controlled to avoid over-coarsening during alloy forming and operation. Such coarsening can lead to a loss of toughness within these alloys [35] and destabilization of martensite. Often there is a competition between MX and M_23_C_6_ formation, with alloy designers seeking to encourage enough fine, high-density MX while M_23_C_6_ remains sufficiently suppressed.

Concurrent with the high-temperature performance, efforts have also focused on improving the alloy toughness at lower operating temperatures, noting progress on this topic in the EUROfusion programme [36]. UKAEA’s 5 years NEURONE (NEUtron iRradiatiOns of advaNced stEels) programme has brought together 11 academic and industrial organizations in the UK to develop a steel grade that might be useful to STEP iterative design. The programme addresses alloy development, processing, manufacturability (including joining), irradiation performance and routes towards qualification as its key objectives. Initially, NEURONE has focused on the optimization of alloy chemistry and refinement of thermomechanical treatment (TMT) process parameters (table 1). The NEURONE alloy chemistry has sought to increase concentrations of elements which result in the formation of thermodynamically stable, nanoscale second phase precipitates. The increase of vanadium and/or titanium allow a greater proportion of stable MX carbides/nitrides to form. The irradiation-induced clustering of Mn and Si is addressed through reduction of both elements to mitigate these issues. In tandem with the theoretical design, the programme has worked with the UK industry to understand what realistic baseline levels of elements are probably to be achieved using existing electric arc furnace facilities. This is to ensure that an alloy is not designed which cannot be reliably replicated at industrial scale later.

**Figure 2.**
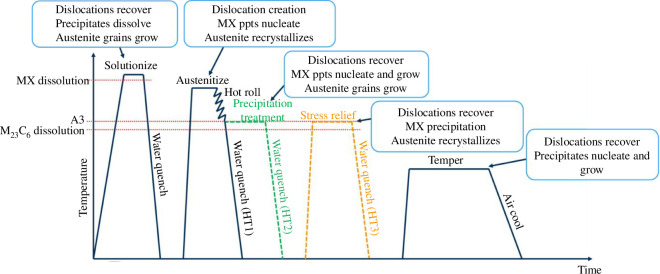
TMTs used within the NEURONE programme. The green dashed line represents a variant of the original heat treatment 1 (HT1), introducing a precipitation treatment step at the A3 temperature (HT2). An additional stress relief step within a third heat treatment variant (HT3), shown as an orange dashed line, is introduced to encourage a refinement of the prior-austenite grains and improve alloy toughness. Actual heat treatment parameters are not shown for confidentiality reasons.

The TMT process being developed has sought to exploit multiple rolling passes alongside adapted heat treatments to refine the microstructures developed for high-temperature operation. Figure 2 demonstrates schematically the types of heat treatments used to produce ARAFM variants. The initial heat treatment used (HT1) is shown in the solid black line, consisting of a simple solution treatment, ensuring that all elements are in solution and that any precipitates are dissolved. This is followed by austenitization (above the A3 temperature), whereby austenite recrystallizes, and MX precipitates can begin formation. Hot rolling and a quench step follow next. This step introduces a high dislocation density into the material, refining the martensitic lath structure which forms upon quenching and enabling precipitation of the fine MX particles at these dislocation sites. Finally, a temper step is carried out, recovering the dislocations introduced earlier, which softens the base martensitic matrix, leading to the formation of tempered martensite. This stage also allows precipitates to fully nucleate and grow.

Figure 2 also shows how HT2 is introduced as an additional precipitation treatment step. This allows for the extended evolution of MX precipitates, while the alloy is held at a temperature above the M_23_C_6_ dissolution temperature. This allows excessive growth and coarsening of the M_23_C_6_ to be avoided. A third heat treatment (HT3) incorporates the precipitation treatment of HT2 and adds a stress relief step, carried out close to the A3 temperature. This step was added to assess the effectiveness of an additional stage after quenching to recrystallize austenite, leading to a PAG size reduction. This refinement should increase the toughness of the alloy, particularly under LTHE scenarios, as evidenced by development and testing of the SCK-CEN Eurofer97 derivative ‘Alloy J’ [30,36,37]. The next phase of the NEURONE programme is to conduct mechanical tests of these alloys at low temperatures to understand the effectiveness of these microstructural optimizations.

## 4. Materials assurance

The STEP programme is not the first time where designers have deployed materials in unchartered territory. The Challenger Space Shuttle and UK Magnox fission plants are two examples where operation in extreme, untested environments was required and where structural integrity assessment necessitated innovative approaches [38]. Consensus on the means for materials assurance within the fusion community is closely based on methods developed over decades of experience within the fission industry for obvious reasons, prescribing compliance with codes and standards, e.g. RCC-MRx [39] for ITER, and the development of fusion specific criteria and sections [40].

The codes specify ‘Design by Rule’ and ‘Design by Analysis’ methodology for structural integrity assessment and the requirements for the provision of materials test data are, rightly, onerous but are also resource intensive. The fusion community has a growing reliance on fission-based materials test reactor (MTR) irradiations to address this, which immediately raises further questions and concerns regarding (i) the differences between the DT neutron and fission neutron energy spectra means that MTR irradiation cannot represent the transmutation anticipated in fusion [41,42]; (ii) cook and look irradiations are not suitable for time-dependent failure and failure owing to C-type loading, such as creep and fatigue which are arguably more probable failure modes for in-vessel components [43,44]; and (iii) the availability of suitable MTRs is limited and reduced owing to barriers associated with geopolitical crises, the general ageing of the global MTR fleet and lack of investment for replenishment. In addition to the challenges associated with exposing materials to a representative environment, the codes also clearly specify the testing of materials that are representative of the manufacturing route used for plant construction. This approach requires sequential materials selection, supply chain and manufacturing development and finally qualification, which places considerable constraints on STEP design (e.g. towards the selection of conventional materials rather than advanced options such as those described in the above section on high-temperature steels) to deliver within the ambitious timeframe of the project.

### (a) Design by fundamentals: Crystal plasticity modelling

STEP must take an alternative approach to structural integrity assessment. This approach must satisfy engineering requirements in the absence of a comprehensive database of material properties; thus, predictive models and advanced simulation techniques will be required. The unprecedented conditions of loading and radiation damage anticipated in STEP require that new models must be developed to:

— Be relevant to engineering requirements, based on failure mechanisms such as those defined in design codes.
— Capture the fundamental mechanisms that are specifically relevant to material behaviour and failure within an irradiation environment.

The Design by Fundamentals (DbF) project was recently initiated within the programme to develop engineering relevant, predictive models which support an understanding of the effects of fusion-relevant irradiation on materials. To satisfy requirement (a), it is imperative that models are developed at a scale which is relevant to material failure within STEP components, while accounting for the nanoscale origins of radiation damage. Deviating from the multi-scale modelling (bottom–up) approach which has commanded the most attention in fusion to date [42,43], we propose that modelling at the meso (microstructural) scale of metals and alloys is most appropriate to satisfy (a) [45–49]. The approach is ‘top–down’ with mesoscale modelling offering engineering relevant insight adjacent to established finite element simulation techniques at component length scales. This overcomes the risks associated with the propagation of error within the bottom–up approach [50,51], by avoiding necessary transfer of information across several length scales and modelling techniques. DbF primarily focuses on the use of targeted experiments to inform physically based constitutive models that can simulate the critical deformation mechanisms which ultimately lead to failure. This top–down approach will extract the physical insights from lower (e.g. atomistic) scale modelling that is accessible to the community today but avoids the impossibly huge development required for a holistic bottom–up approach, from atom to component, necessary for engineering prediction.

**Figure 3.**
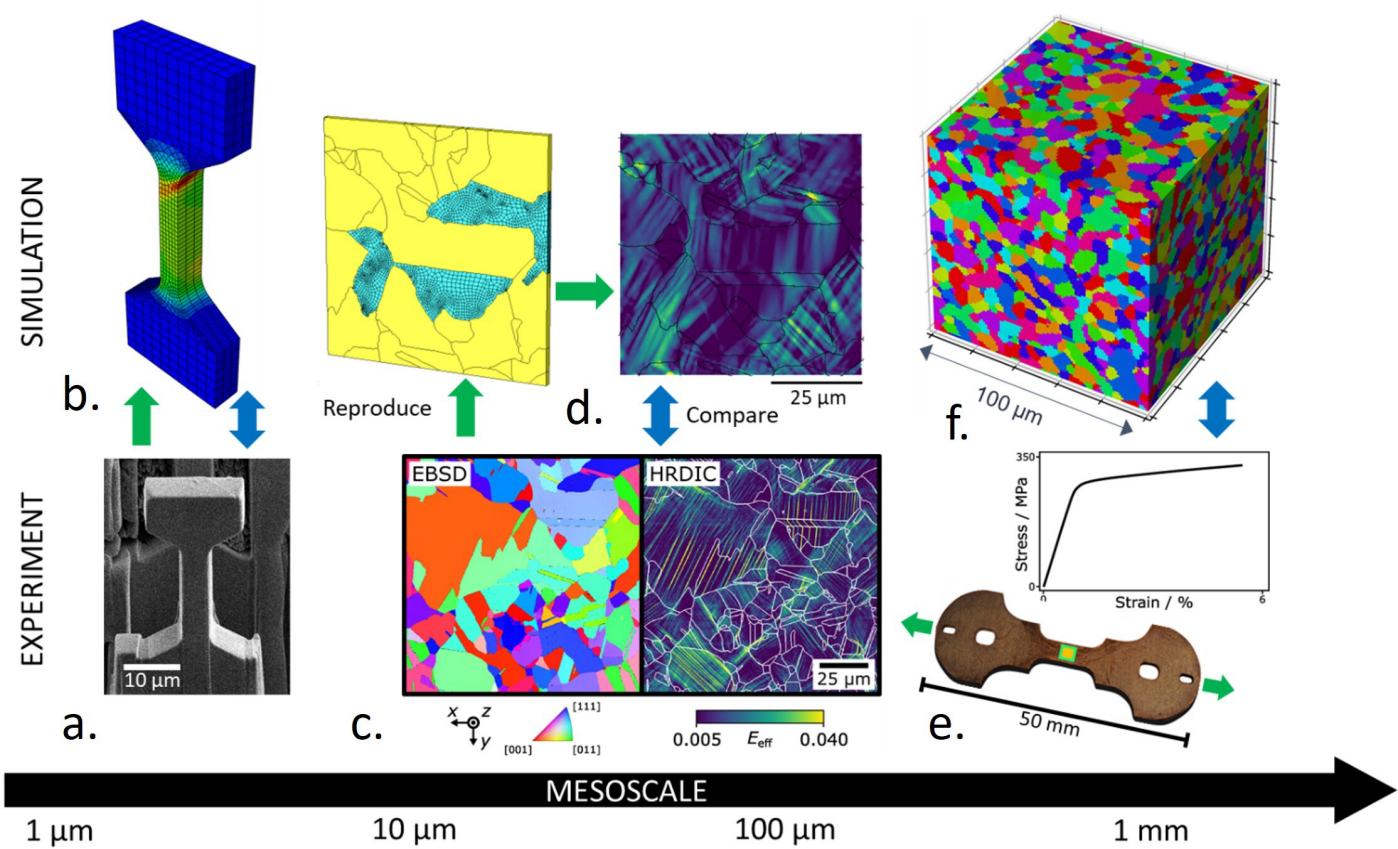
Schematic of the DbF strategy, highlighting early results including (*a*) micro-tensile test specimen and (*b*) corresponding CPFEM simulation output, (*c*) high-resolution digital image correlation (HRDIC)–electron backscattered diffraction (EBSD) data of deformed CuCrZr microstructure and (*d*) corresponding CPFEM model, (*e*) miniature CuCrZr tensile specimen with stress–strain data and (*f*) developed representative volume element (RVE) for simulation of bulk behaviour.

As shown in figure 3, DbF is a multi-disciplinary project focused on the systematic calibration and validation of mechanical models, underpinned by crystal plasticity finite element methods (CPFEM) [52] and related techniques to fully capture deformation mechanisms including plasticity, creep and fracture. These techniques have already been impactful in other sectors, e.g. aerospace [53]. The strategy aims to test the simple hypothesis that if a model can capture the deformation at the microstructural scale, which is primarily governed by elasticity, dislocation-mediated plasticity, and failure in the form of crack initiation and growth, it can predict material response against the variety of failure modes described in the design codes. In the absence of a fusion neutron source capable of exposing materials to a representative fusion environment, surrogate sources of irradiation are being used for experiments within DbF, which isolate and reproduce important aspects of fusion-relevant irradiation. Examples of DbF efforts include the use of proton beamlines to irradiate specimens while under mechanical loading to measure irradiation creep rates, as well as dual beam irradiation to measure the influence of transmutation products such as He on mechanical behaviour. Our initial focus has been on irradiation-induced plastic strain localization. Here, we have demonstrated the ability of CPFEM to reproduce experimentally measured strain softening and localization in irradiated Zircaloy-4 [54], using a consistent mechanistic formulation for slip and defect interaction and annihilation. This deformation behaviour acts as a precursor to several failure modes including plastic collapse, premature intergranular fracture and irradiation-assisted stress corrosion cracking [55,56]. The promising results from this early work have resulted in a long-term strategy to develop models towards property prediction and the initial output has delivered essential foundational work.

Point (b) is specified to address the challenges/limitations described above, i.e. representation of the fusion irradiation environment, noting that testing material in an unirradiated condition is comparatively trivial. During these early, conceptual phases of STEP, charged particle irradiation has been used as a practical surrogate to fusion neutron irradiation and the focus on the demonstration of techniques on ferritic-martensitic steels and CuCrZr alloys as potential candidate structural materials. Irradiations have primarily been conducted using proton beamlines owing to their versatility and large penetration depths [57,58], which enables through thickness irradiation of miniature tensile specimens for post-irradiation experimentation and *in situ* testing. As suggested above, a bespoke rig has been developed for the latter, which is currently being used to investigate and measure irradiation creep.

State-of-the-art techniques to test materials are essential to unlock information on deformation mechanisms, which is itself essential for the development of representative models. Nanoindentation and micromechanical testing are used for site-specific testing of microstructural features, such as single crystal testing within a polycrystalline sample, grain boundaries and irradiated surface layers in the case of charged particle irradiation. Micromechanical test data are used to isolate features and parameterize constitutive formulations such as within crystal CPFEM, which accurately capture the observed deformation mechanisms with a unique solution [59,60]. Full characterization of the tested microstructure is essential for linking to mechanics in the simulations and includes EBSD for crystal microstructure (modelled explicitly) and (planned) transmission electron microscopy for lattice features which are homogenized and modelled intrinsically in the formulations. Deformation on larger length scales is measured by HRDIC strain mapping coupled with EBSD measurements; these provide rich data on the key deformation mechanisms following mechanical testing supporting parameterization and validation from full-field measurements [61]. These tests are facilitated by bespoke surface patterning techniques which were recently developed for irradiated samples [62]. Finally, simulations on RVEs are used to provide a means of *in silico* mechanical testing for bulk mechanical property predictions and sub-modelling for thermo-mechanical simulations of components. A bespoke in-house code for Monte-Carlo simulations has been developed to produce dedicated RVEs, which are based on customized Metropolis algorithms [63]. These methods include quantitative representativeness assessment and qualification of the grain structure models against experimental characterization (EBSD) of STEP relevant materials.

The DbF project aims to depart from current methods for structural integrity assessment of FOAK plant, such as STEP, which is anticipated to expose components to unchartered, untestable environments. On one hand, this is a radical approach born out of constraints associated with the highly ambitious timeframes and resource limits of STEP; on the other hand, it could be argued that this is simply a placement of higher emphasis upon theory and modelling, which is complementary to several, more established international plans and efforts. An example of the latter can be taken from the key requirement defined within the EU-DEMO readiness assessment for qualifying as ‘Materials Technology Readiness Level’ (MTRL) 6 within the EUROFusion programme: ‘Modelling to extrapolate from fission/MTR irradiation data to fusion neutron conditions’ [64].

## 5. Active waste mitigation strategies in STEP

Neutrons will be produced from deuterium–deuterium and deuterium–tritium reactions in the STEP reactor, which will lead to the activation of materials [65]. After the reactor is shut down, all STEP waste components will be categorized as set out in policy and defined in UK regulation and need to be managed accordingly [66,67]. Components will be mostly categorized as LLW and intermediate-level waste (ILW) [68]. Previous work on experimental scale powerplants such as EU-DEMO and ITER has shown that the volumes of waste will be increasingly large—a problem if more fusion reactors are built as a plan for managing waste arising needs to be developed [69–71]. Like EU-DEMO, STEP also aims to demonstrate the feasibility of commercial operation, and therefore expose components and materials to high neutron fluxes for longer periods than either ITER or any previous experimental reactor. Since STEP aims to demonstrate commercial operation, it will experience a higher neutron flux and longer operation time. This higher neutron fluence will lead to higher radioactivation of materials and hence could lead to the generation of higher waste volumes compared with ITER or any previous fusion experiment. Waste will arise from all reactor materials, including the different types of structural steels producing long-lived radioisotopes from alloying elements, such as C, Nb, N, etc. [72]. There are also uncontrolled impurities that exist within these materials that can affect waste categorization, such as Co and K. Waste predictions for STEP are determined by combining neutron transport simulations with inventory simulations (e.g. with FISPACT-II) but are also influenced by previous work and literature from DEMO and ITER studies to predict radwaste arising from operation.

To understand the effect of neuron activation, simulations with the nuclear inventory code FISPACT-II using TENDL2019 nuclear data library were performed [73,74]. This model assumed a 1.772 GW of fusion reactor operating in phase-wise operating scenarios for over 2 full power years. Table 2 defines the UK waste categories in activity terms.

**Table 2.** Activity of alpha and beta/gamma radiation defining waste categories.

	LLW	ILW
alpha activity	<4 GBq/tonne	>4 GBq/tonne
beta and gamma activity	<12 GBq/tonne	>12 GBq/tonne

**Figure 4.**
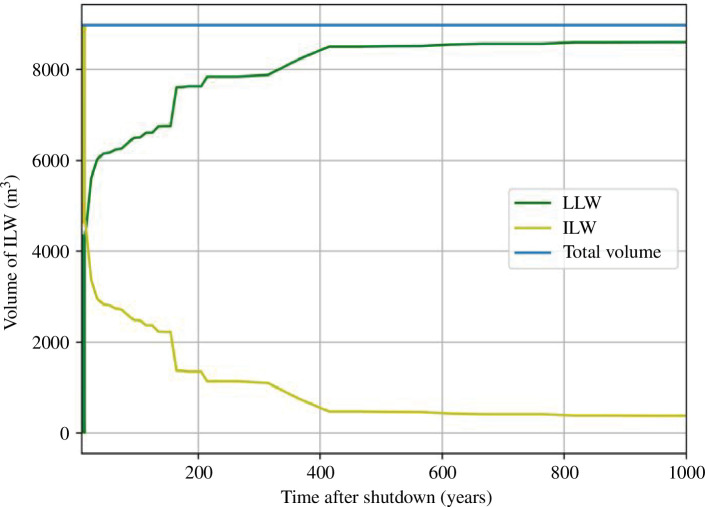
Indicative total waste volume assessment of waste categories for prototype STEP reactor up to 1000 years post-shutdown.

Using the inventory simulation outputs, the different components and materials in the reactor model were attributed to different radioactive waste categories set out in policy and defined in regulation [66]. The volume of waste categorized as ILW decreases greatly within 50 years post-shutdown of the reactor, with the activity of components reducing to below the LLW limit, as shown in figure 4. The volume of waste categorized as LLW increases significantly within the first 200 years, arising from the decay of short-lived nuclides which bring waste that was initially within the ILW category below the LLW limit. However, up to 1000 years post-shutdown, there is predicted to still be significant waste volumes categorized as ILW.

**Figure 5.**
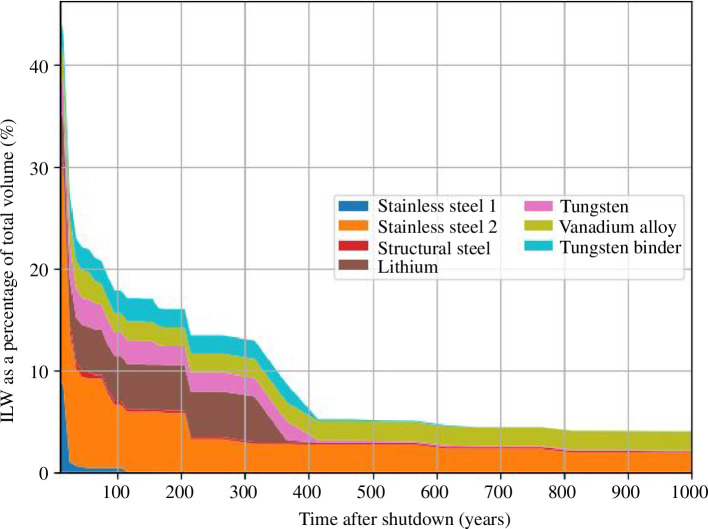
Indicative material-wise illustration of ILW as a percentage of total volume per unit.

To further understand the materials that contribute to ILW, figure 5 depicts the percentage contribution to ILW by the key materials against time after shutdown. At this stage of design, these analyses exclude the coolants (which were included in figure 4) but the STEP programme is not yet in a position to fully qualify coolant flow and volumes. Structural materials like stainless steel, vanadium alloy and tungsten constitute most of the ILW produced. Volumes of material that could be categorized as ILW reduce dramatically within decades and within 100 years less than 20% of the total waste is predicted to be considered ILW for disposal (excluding coolants).

Structural materials are the majority of ILW with nuclides such as 3H, 63Ni, 94Nb and 14C contributing to the ILW categorization. These can have varying effects on time of disposal based on half-lives, e.g. 3H has a half-life of 12.33 years whereas 14C has a half-life of 5730 years. Long-lived nuclides such as 94Nb and 14C are among the nuclides that are responsible for materials not reaching the LLW limit within 1000 years post-shutdown. The estimation of the waste streams for STEP changes with each design iteration as there is a continuous exploration of various design options. To facilitate these studies and to enable agile model optimization, a waste database infrastructure has been developed to store information about each model in a centralized location and format. This approach will allow for querying based on the different requirements and will be used to allow for waste performance comparisons across STEP models and design scenarios. This can eventually influence final STEP designs by assessing each model in a regularized information format, accessed through a fixed framework.

Waste measures should follow a hierarchy to first consider avoidance, reusing and recycling before disposal. Waste assessments have directly motivated research and development into techniques to reduce activity and improve the handling of waste materials which STEP is pioneering. These include the development of low-temperature tritium permeation barriers to aid the containment of tritium-contaminated waste and the exploration of detritiation factors using acid etching. Tritium permeation research aims to measure permeation on a range of representative materials to support material selection and waste modelling. Most of the tritium lies in the surface layer of most materials; acid etching can be used to remove these surface layers to facilitate the recycling of bulk waste materials. These research areas are currently being developed in conjunction with STEP models for post-shutdown processing options. Additionally, the STEP programme is developing oxidation control techniques to mitigate the potential radiological hazard that would arise, for example, from the mobilization of radioactive W-oxides that could be produced in accident scenarios (loss of vacuum) or during maintenance activities.

Waste assessments are necessary to understand the handling, treatment and disposal options of the high volumes of waste predicted to arise from STEP operations. ILW mostly originates from structural materials that have been irradiated at high neutron flux leading to the production of long-lived nuclides. These radionuclides can remain in significant concentrations for 1000 years or more post-shutdown, which is undesirable for fusion, where the target is a low waste legacy and high environmental sustainability. R&D projects are in progress to explore avenues of reducing ILW through reduced activation compositions in structural materials. The results gained from each iteration of the STEP model are valuable information to the programme and wider fusion communities, especially when developing waste strategies. Therefore, the waste database developed has been immediately implemented with the potential to transform how STEP compares models across time.

## 6. Closing thoughts

The STEP environment will include high magnetic fields, mechanical loads, high heat fluxes, erosive plasma interaction, hydrogenous gases and corrosive coolants [75] (in series). In addition, in-vessel materials in STEP are anticipated to be exposed to peak dose rates of 10^−6^ dpa/s or approximately 0.1 dpa per full power day. Given the striking degradation of properties upon exposure to fission neutron doses of this magnitude (e.g. hardening and loss of ductility in most alloys [76–79]), the effect of fusion neutrons throughout the service life must be understood. However, while facilities to test materials under thermal, electromagnetic and mechanical loads exist [80–82], even in combination [83], test capability for fusion representative neutron loads do not. The STEP materials team has therefore constructed an irradiation strategy that stitches together multiple uses of ions, protons and neutrons, in combination with transmutation proxy methodologies, to create a combined view of the interdependent displacement and transmutation effects in plant-relevant materials. This irradiation strategy must support (and be informed by) the full materials life cycle: downselection, development, assurance and end-of-life strategies.

In this article, we have briefly listed examples of current work in all four of these areas, focusing mainly on structural materials. We have discussed how the materials downselection is oriented in plant power trade-off space, outlined the development of just one materials family (steels), described our 'Design by Fundamentals’ approach for structural integrity assessment of FOAK plant and considered some of the waste mitigation routes intended to make STEP operations as sustainable as possible.

## Data Availability

Data will be accessible through the UKAEA Open Data Register.
